# Notes and News

**Published:** 1892-05-28

**Authors:** 


### Notes and News.

Collected and Communicated.

The new buildings of the Poplar Hospital for Accidents, Blackwall, are to be finished in November. These buildings are for men only, and it seems to be a mistake that there no ward for the reception of women and children has also been provided. In such a neighbourhood the local aid is necessarily small, and special appeals are absolutely necessary. It may be said in favour of the institution that it has never exceeded its income, and that the working classes contribute a fifth of its income. It is to be hoped that wealthy localities will come forward to aid this most beneficial charity, which is situated in the midst of constant scenes of accidents from the docks and great iron works.

A letter appeared in the *Daily News* last week, written, the writer states, to call attention to the present defects in ambulance conveyance. The picture he describes is truly heartrending, and as he himself was a participator in the discomforts depicted, the narrative has all the vividness of reality. "After waiting at the Gray's Inn Road Workhouse over three hours, in a starving condition, and with the commencement of rheumatic fever upon him, the narrator commenced his journey to the Holborn Infirmary. His companion on starting was a poor woman suffering from frost-bitten feet, and crying with pain. The jolting he describes as frightful, worse than that of a country cart. His sojourn in the van lasted two hours and a half, during which time other patients were collected, several on stretchers, until the van was sadly crowded." Now the writer of this account, who signs himself "W. Williams," protests on two scores. One is that the van is too long *en route*, and the other that it is not large enough. All will agree with him, I imagine, that a journey thus of over two hours is a very serious evil, but most will see that his complaint as to the size of the conveyance, if remedied, would only tend to increase this evil, by making it possible to admit more patients. More, not larger, ambulance carriages are needed, and these actually are increasing in number as quickly as the funds will allow. The plea for india-rubber tyres for such conveyances is a reasonable one, and will, it is hoped, be attended to.

The Homes for Little Boys at Farningham and Swanley held their annual fête on Saturday, 7th inst., in delightful weather. A large company was present, and witnessed the crowning of the May Queen. From a physiological point of view the musical drill and the various athletic exercises were eminently worthy of approval. The homes well deserve liberal support.

Monsieur Ouvre, Deputy Consul-General of the Seine-et-Marne, has offered the county the beautiful old Abbey of St. Severin, at Chateau-Landon, of which he is the proprietor, as an almshouse for aged men, as a free gift. The Abbey is beautifully situated for the purpose, and the General Council have gratefully accepted the offer, and will set about organising the scheme.

The new Convalescent Home for Women is to be opened at Great Wakering, it is hoped, in July. Four hundred pounds is the sum estimated as sufficient to cover the working expenses of the Home when once established. So small a sum should be easily forthcoming. Those who prefer to contribute in kind are invited to help by gifts of books, furniture, and other useful articles. The institution is to be managed by a Ladies' Committee, and will be entirely undenominational.

From our babyhood to the days of second childhood our teeth cause to most of us a considerable expenditure of patience and of money, but how few of us when paying our dentist's bill cast a thought on the thousands of people in London alone, for whom skilled attention is an impossible luxury? The question of securing proper care for the teeth of the children of the poor has been discussed by the British Dental Association for some years, and a practical step towards the attainment of this end has now been taken and valuable statistics recorded respecting the pupils at the Central London District Schools at Hanwell. Out of 1,000 children examined by Messrs. Denison Pedley and Sydney Spokes, in only 137 mouths was the dentition sound; there were 1,119 temporary teeth which required stopping and 745 needing extraction; of the permanent teeth 1,222 required filling and 271 extracting. These alarming figures go far towards preparing us for the advice embodied in this valuable report, that a duly qualified dental surgeon should be appointed to such a school, and we are glad to find the suggestion has been carried out by the Board of Management. Our readers will cordially agree in the wisdom of this step in the right direction, and we hope to see the day when all district and parochial schools shall be similarly looked after and the dentist considered "a necessity, not a luxury," as the medical press headed its article the other day. Attention has also been called to the present neglect of the teeth of our soldiers, especially the recruits, and this is assuredly an unpardonable omission. Some of the naval surgeons are already making progress in the right direction for they are studying this important subject both at general and dental hospitals with a view to giving instruction to other naval surgeons as well as for the permanent benefit of those sailors under their own special care.

